# Supplementary material for: MEF2C Directly Interacts with Pre-miRNAs and Distinct RNPs to Post-Transcriptionally Regulate miR-23a-miR-27a-miR-24-2 microRNA Cluster Member Expression
Source: Noncoding RNA. 2024 May 17;10(3):32. doi: 10.3390/ncrna10030032 (PMC11130849; doi:10.3390/ncrna10030032)
Supplement: Supplementary file 1 [file ncrna-10-00032-s001.zip › Mef2c RNPs Supplementary Materials.pdf]

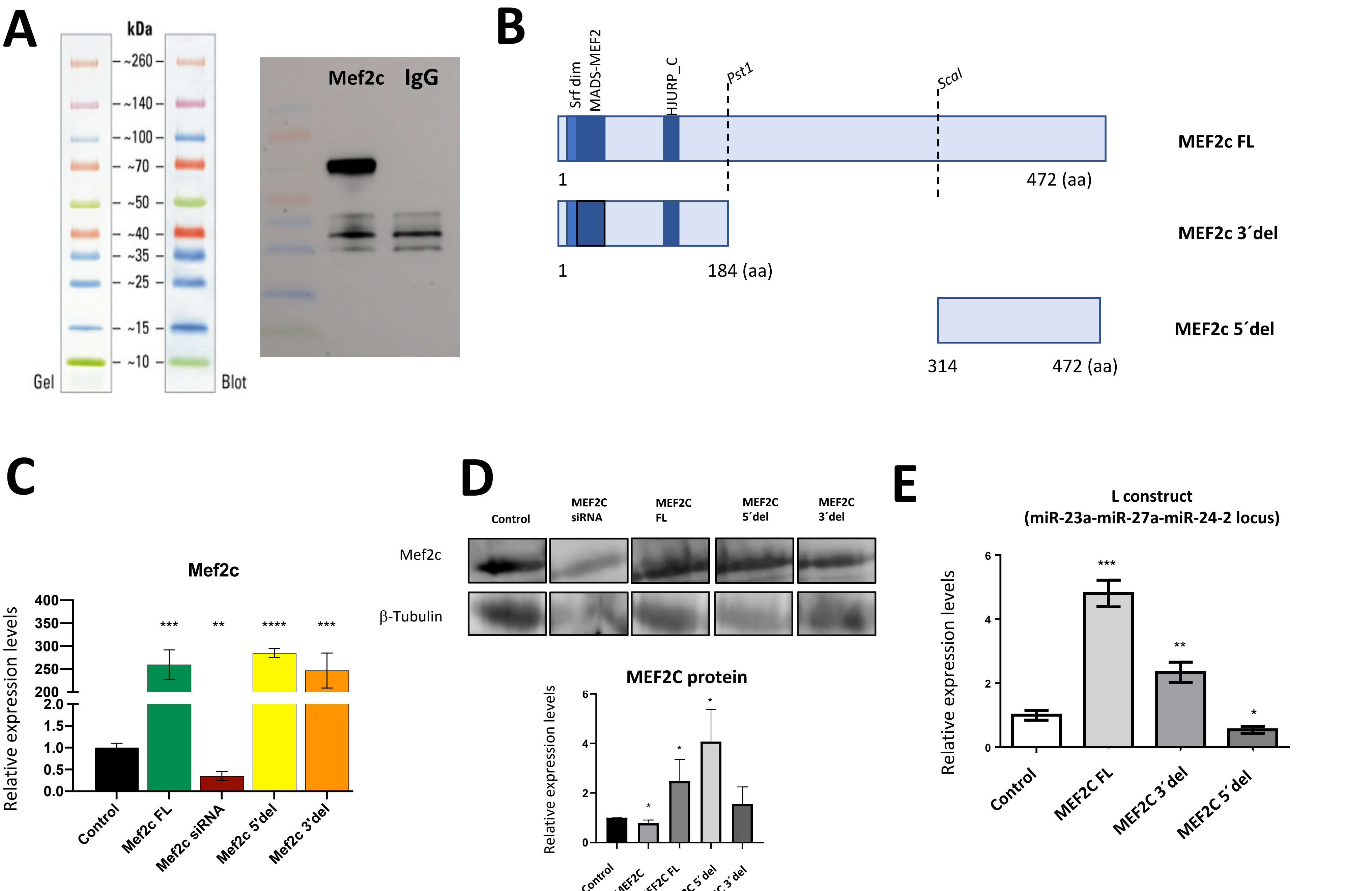

Supplementary Figure 1

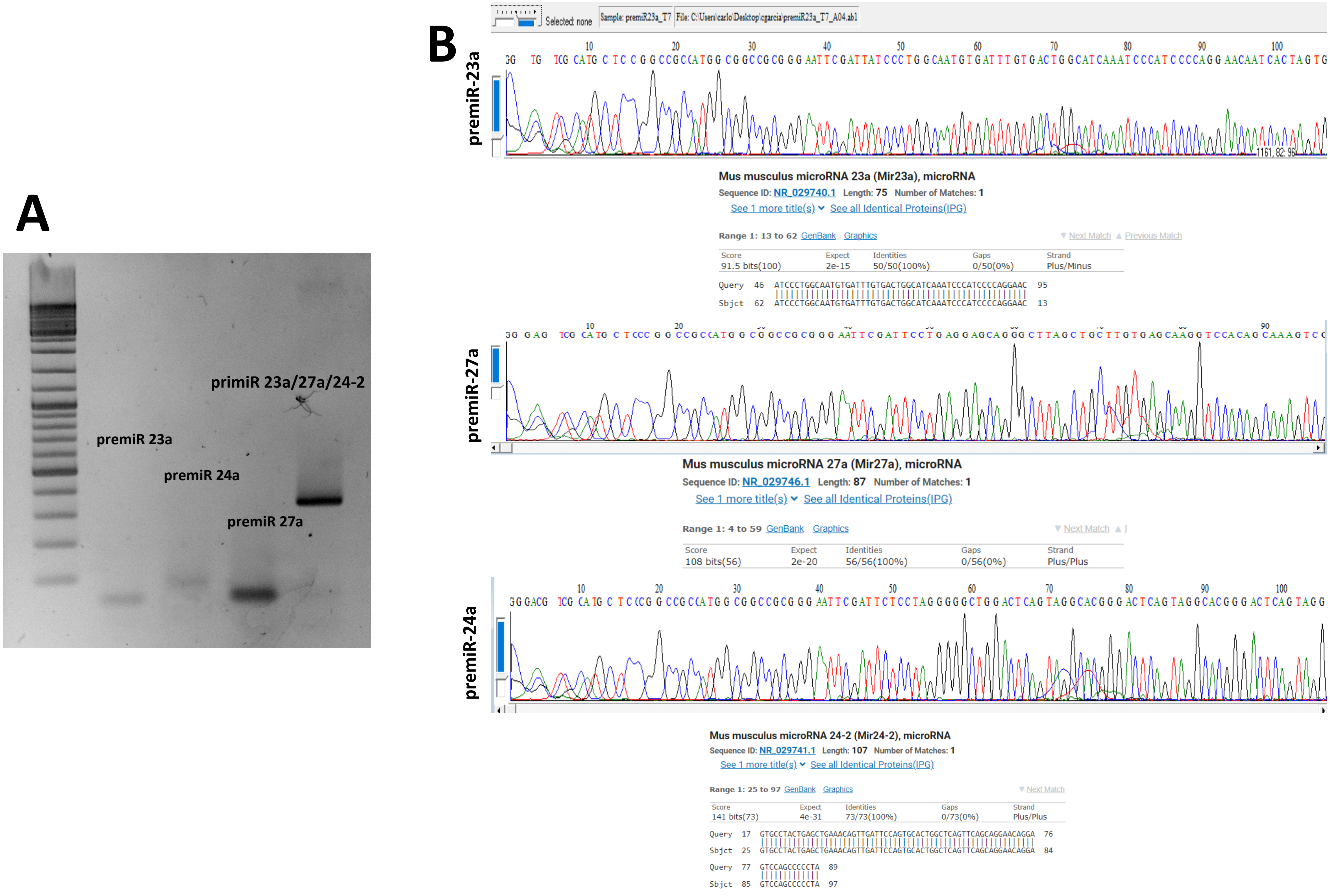

Supplementary Figure 2

miR 23a – miR 27a – 24a-2p

GAGTTTCCTCCTGCCCTTCAGGCTTCTAGGAAGTGGCGCCAGCTGGGGTGAGGTCACTTC
CTCCCTCCAACCGTCCACCTCCCTCAGCTTCCTCTCCATGGCCCCATTTGGCCTGCTTTG
GGCTCAGGGCTGTGGGTGGGGATAGAAGCAGAGTGGGAAGCAGCTGGGAAGGCTCTGCAG
CCCCTTTGGCCTTAGGGCTTCAGGCACCTCCCGCTTTGTAGGGCTGGGGTAGAGGCACTG
CTGGGTGCCCAGCATCCAGCAGCCTCGCTCCAACCTTCCTACGGATCGATGCTCCTCTTT
TTTGGGAATGCTTCTTCCCTCTTGGAAGTAGAGGAGGGCTAGGGTGTGGATCAGCAAGCT
GAGTTTAGATTCTGTCCCCAGCACCACTTAAACTGTGTGTGGTGAGGTGTACCTATAATC
CTAGACTCCAGAGGTAGAGGCAGGAAGAACTGGTGCATTCGGAAACCTTGTGTTCAGCTA
TGTGAGACCCAGCCTGGTCAAGATAGGCAGGCAAGCAAGAATGCTCCAATCTCACTGTCT
CTTCTTTCTCTCTTTAGGTGCTACACTCCGCTCCCACCTGCTGCCCCCCTCACTGTGCCT
CGGACGGCTGGGGTTCCTGGGGATGGGA**TTTGATGCCAGTCACAAATCA**CATTGCCAGGG
ATTTCCAACTGACCCTGTGCTCTGCCTTGGGGGCTCCTGTCGCCAAGGATGTCTGTCTTG
GGTACTGGGTAACAGAGAAGCCTATCATGACAACTGGCCTGAGGAGCAGGGCTTAGCTGC
TTGTGAGCAAGGTCCACAGCAAAGTCGTGTTCACAGTGGCTAAGTTCCGCCCCCTGGACC
CCATCTCCTCAGGCCGCTGCTGCCCACCCTGCTCTGCCTCCTGGCTGGCAGGGCTCTGCC
TCTCTCCGGGCTCCGCCTCCCGTGCCTACTGAGCTGAAACAGTTGATTCCAGTGCACTGG
CTCAG**TTCAGCAGGAACAGGAGTCC**AGCCCCCTAGGAGCTGGCAACCCTGCAGAATGTCT
GCCGTGAGCAAGACCTGGAAACAATGGGGTAATTAGTCCAACCTAAGCCCCGTTGTCTGC
TGGAGGCTCAGCCTCTAAGCCTGCATCTGGGTGTCCACCTTGAGATTTCTGTGGTTCCCC
AAAGCAGATAGATGGGCGGGTAACCCTTGCATTAAAAGCCAGTTATGTCCCCTCAGGGCC
TGGAAGCCCTTCTTTTCCTAAGTCTGGTGGGGCTCAGGCACCACCCCTTATACATACCAT
CTTTGAAGTCAGTTA

premiR-23a

premiR-27a

premiR-24a

PrimiR 23a-27a-24a

Name Primer	Sequence	Amplicon size
Pre_23a_F	GTTCCTGGGGATGGGATTT	53 pb
Pre_23a_R	ATCCCTGGCAATGTGATTTG	
Pre_27a_F	CCTGAGGAGCAGGGCTTAG	65 pb
Pre_27a_R	GAACACGACTTTGCTGTGGA	
Pre_24a_F	CCTCCCGTGCCTACTGAG	90 pb
Pre_24a_R	CTCCTAGGGGGCTGGACTC	
Pri_miR_F	TTTGATGCCAGTCACAAATCA	387 pb
Pri_miR_R	GGACTCCTGTTCCTGCTGAA	

Supplementary Figure 3

Gene	Sequence
Mm_Gapdh	Fw: GGCATTGCTCTCAATGACAA
	Rv: TGTGAGGGAGATGCTCAGTG
Mm_ Mef2c	Fw: TGGTTTCCGTAGCAACTCCT
	Rv: AGTTACAGAGCCGAGGTGGA
Mm_Adar1	Fw: TGACCAGAGATGGGAAAGCA
	Rv: ACCAGTTGACGCTTGTTTCC
Mm_Ddx5	Fw: ATCTTGATGAGCTGCCCAAA
	Rv: GGTTTTGGACAGTTGTGACCT
Mm_Ddx17	Fw: CCTCATTGCCACGGATGTAG
	Rv: TATAGGCAGTGCCCTTGTTG
Mm_hnRNPa1	Fw: TGAAGGGAGGAAACTTTGGA
	Rv: TGCCATAGCTACTGCTGCTG
Mm_hnRNPa2b1	Fw: GGCAGTCTGGAAAGAAAAGAGG
	Rv: GGACTTCCTGCATTTCTTGTCT
Mm_hnRNPa3	Fw: GGAGGAGGAGGCTATGATGG
	Rv: CTTCCACCAAAACTGCCCC
Mm_Ksrp	Fw: ATCGGAGGTGATGCTGCTAC
	Rv: CCTGGGAAGCCAGTTTCTT
Mm_Pre-miR23	Fw: GTTCCTGGGGATGGGATTT
	Rv: ATCCCTGGCAATGTGATTTG
Mm_Pre-miR27	Fw: CCTGAGGAGCAGGGCTTAG
	Rv: GAACACGACTTTGCTGTGGA
Mm_Pre-miR24	Fw: CCTCCCGTGCCTACTGAG
	Rv: CTCCTAGGGGGCTGGACTC
hsa-miR-23a, LNA™ PCR primer set	AUCACAUUGCCAGGGAUUUCC
hsa-miR-27a, LNA™ PCR primer set	UUCACAGUGGCUAAGUUCCGC
hsa-miR-24.2, LNA™ PCR primer set	UGGCUCAGUUCAGCAGGAACAG

siRNA gene	Sequence
siRNA Mef2c	Sense 5’ GGGUCGAUAUGCAUUAUAU UU
siRNA Adar1	Sense 5’ GAUUGUCGAACAACAGGAA UU
siRNA Ksrp	Sense 5' CACCGUGAAUAACAACACU UU
siRNA HnRNPa3	Sense 5' GGUUGUUCAUAAGGGCUAU UU
siRNA HnRNPa2b1	Sense 5’ GUCUCAUACACAUACCCGU UU
siRNA HnRNPa1	Sense 5’ GUCUGAUCAUGACGCUGA UU
siRNA Ddx17	Sense 5’ GGUACUUAAUGAGUUCCGA UU
siRNA Ddx5	Sense 5’ GUUAGGUUCACUAUGGGCU UU

Table 1. List of the primers and siRNA sequences.
